# Abnormal dynamic functional connectivity during fear extinction learning in PTSD and anxiety disorders

**DOI:** 10.1038/s41380-022-01462-5

**Published:** 2022-02-10

**Authors:** Zhenfu Wen, Jeehye Seo, Edward F. Pace-Schott, Mohammed R. Milad

**Affiliations:** 1 Department of Psychiatry, New York University Grossman School of Medicine, New York, NY USA; 2 Department of Psychiatry, Massachusetts General Hospital, Charlestown, MA USA; 3 Harvard Medical School, Charlestown, MA USA; 4 The Neuroscience Institute, New York University Grossman School of Medicine, New York, NY USA; 5 Nathan Kline Institute for Psychiatric Research, Orangeburg, NY USA

**Keywords:** Psychiatric disorders, Diagnostic markers

## Abstract

Examining the neural circuits of fear/threat extinction advanced our mechanistic understanding of several psychiatric disorders, including anxiety disorders (AX) and posttraumatic stress disorder (PTSD). More is needed to understand the interplay of large-scale neural networks during fear extinction in these disorders. We used dynamic functional connectivity (FC) to study how FC might be perturbed during conditioned fear extinction in individuals with AX or PTSD. We analyzed neuroimaging data from 338 individuals that underwent a two-day fear conditioning and extinction paradigm. The sample included healthy controls (HC), trauma-exposed non-PTSD controls, and patients diagnosed with AX or PTSD. Dynamic FC during extinction learning gradually increased in the HC group but not in patient groups. The lack of FC change in patients was predominantly observed within and between the default mode, frontoparietal control, and somatomotor networks. The AX and PTSD groups showed impairments in different, yet partially overlapping connections especially involving the dorsolateral prefrontal cortex. Extinction-induced FC predicted ventromedial prefrontal cortex activation and FC during extinction memory recall only in the HC group. FC impairments during extinction learning correlated with fear- and anxiety-related clinical measures. These findings suggest that relative to controls, individuals with AX or PTSD exhibited widespread abnormal FC in higher-order cognitive and attention networks during extinction learning and failed to establish a link between neural signatures during extinction learning and memory retrieval. This failure might underlie abnormal processes related to the conscious awareness, attention allocation, and sensory processes during extinction learning and retrieval in fear- and anxiety-related disorders.

## Introduction

Conditioned fear inhibition is achieved by the repeated presentations of the conditioned stimulus in the absence of the unconditioned stimulus. This fear extinction process is critical for fear reduction in the aftermath of trauma exposure or when exposed to fear- and anxiety-inducing stimuli. Failure to appropriately extinguish fear could contribute to the maintenance of anxiety-related symptoms, which is thought to characterize posttraumatic stress disorder (PTSD) and anxiety disorders [1, 2]. Pavlovian fear extinction is a widely used translational model for studying the mechanisms of fear extinction [3–5]. Rodent and human studies suggest that fear conditioning and extinction engage a network of brain regions including the ventromedial prefrontal cortex (vmPFC), dorsal anterior cingulate cortex (dACC), hippocampus, amygdala, and insular cortex [3, 6]. This ‘fear network’ is extensively involved in threat-detection, regulating defensive reactions, and emotion processing [7]. Impaired activations within the ‘fear network’ have been reported in various psychiatric disorders using fear-related paradigms as well as other emotion-provoking tasks [8–10]. LeDoux and Pine recently questioned the traditional view regarding the contribution of the ‘fear network’ to the subjective feeling of fear [11]. That is, the so-called ‘fear network’ is mostly composed of brain regions essential for the detection and responding to threat. As such, higher-order cognition, attention, and sensory systems ought to be engaged and interact with the threat detection network to enable our conscious feeling of being afraid and anxious.

Most human neuroimaging studies have yet to establish a connection between cognitive/attention networks and the subjective measures of fear and anxiety. Recent studies started to emerge in support of the LeDoux-Pine concept. For example, meta-analyses revealed the engagement of multiple cortical regions during the fear conditioning and extinction tasks [12], but these activations have not been linked to any subjective measures of fear and anxiety. Activations across well-defined systems including the default mode and salience networks have been reported to be engaged during fear-related tasks [13]. Using a population of healthy controls, we recently showed that dynamic functional connectivity (FC) across distributed brain regions, especially within the default mode and frontoparietal control networks, gradually increased as extinction learning progressed [14]. These results support the idea that large-scale brain systems- involved in attention control, conscious awareness, and sensory motor function are engaged during threat extinction learning and fear regulation. Nonetheless, the relevance of these broad changes in FC to subjective anxiety and trauma related metrics have not been assessed in individuals with psychiatric disorders.

In this study, we examined the dynamic changes of large-scale FC across extinction learning in healthy individuals and in patients diagnosed with anxiety disorders or PTSD. We estimated whole-brain connectivity of participants during different timepoints of extinction learning, compared the FC change between groups, and evaluated the relevance of extinction-induced FC changes to various fear- and anxiety-related clinical metrics. Based on our previous study [14], and given evidence in the literature showing deficient extinction learning and memory retrieval in patients with anxiety and PTSD [3, 9, 15], we hypothesized that individuals with anxiety disorders or PTSD would show less FC increase than healthy controls during extinction learning that is specific to the conditioned stimuli. We also predicted that abnormal FC during extinction learning would be related to brain activations and FC during extinction memory recall, and would associate with symptom measures across all participants.

## Methods

### Participants

We analyzed data from a total of 338 individuals (see Supplementary Methods). Of those, 77 were healthy controls (HC), 91 were diagnosed with anxiety disorders (AX), 81 were diagnosed with PTSD, 89 were trauma-exposed non-PTSD controls (TENC) (Supplementary Table S1). Some results from these data have been published elsewhere with a different focus [16–18]; the current results are novel and have not been previously published. All procedures were approved by the Partners HealthCare Institute Review Board of the Massachusetts General Hospital, Harvard Medical School. All participants provided written informed consent before they participated in the study.

### Experimental procedure

Participants underwent a validated 2-day fear conditioning and extinction paradigm in the fMRI scanner [19, 20]. Details of the paradigm and fMRI acquisition are provided in the Supplementary Methods. Briefly, participants completed the fear conditioning phase on day 1. During conditioning, they were presented with three cues (conditioned stimulus, CS), two of which were partially reinforced with a mild electric shock (CS + ) and the other was not reinforced (CS−). Fear conditioning was followed by an extinction learning phase, where one of the CS + and the CS− were repeatedly presented in the absence of shock. Twenty-four hours later (day 2) participants underwent an extinction memory test. During this phase, they were presented with all three cues: the extinguished CS + (CS + E), the unextinguished CS + (CS + U), and the CS−, to assess their extinction memory.

### Dynamic functional connectivity

As in our previous study [14], we estimated the dynamic FC using a jackknife procedure, such that we could measure the relative difference in FC at a specific trial compared to other trials [21]. We divided the whole-brain into 432 regions, including 400 cortical regions [22] and 32 subcortical regions [23]. We then combined the beta-series correlations method [24] and the jackknife correlation method to estimate task-based FC [14, 21], which resulted in a 432×432 symmetric matrix for each trial. We reordered FC matrices according to CS type (CS + and CS−) and presentation time (from the first to the last CS trial) during the experiment, evenly divided the trials of each CS type into 4 time-blocks (from time-block 1 to 4, representing early to late extinction learning) and averaged trials within each time-block. We thereafter obtained 4 FC matrices during CS + and 4 during CS− processing for each participant. See Supplementary Methods for additional details.

### Statistical analysis

We previously reported that the FC gradually increased from early to late extinction learning in a HC population. This increase was specific to the CS+ and predicted the magnitude of extinction memory [14]. Given that prior studies reported deficient extinction learning and memory retrieval in patients with anxiety and PTSD [3, 9, 15], we hypothesized that the AX and PTSD groups would exhibit deficient FC increase during extinction learning. We specifically examined the FC change during CS + processing from early to late extinction (ΔFC, defined as FC in time-block 4 minus FC in time-block 1). For each connection/edge, we performed a two-sample *t*-test to compare ΔFC between the HC and AX groups or between the HC and PTSD groups, while controlling for age and sex as covariates. We used the Network-Based Statistic (NBS) procedure [25]—a well-validated method for controlling family-wise error (FWE)—to identify network components that showed significant difference between groups. The primary component-forming threshold was set to *p* < 0.001, the significant components were identified with *p*_*FWE*_ < 0.05 using permutation tests (see Supplementary Methods for details).

To test if ΔFC during learning was relevant to the neural signature of extinction memory recall, we conducted cross-phase correlation analysis. In this multiple regression analysis, the independent variable was the mean ΔFC across the identified components, the dependent variable was the brain activation or FC in the extinction memory test (CS + E vs. CS + U, first 4 trials of each type). The age and sex data were included as covariates. Significant activations were identified at a voxel-level *p* < 0.001 and a cluster-level *p*_*FWE*_ < 0.05. Significant network components were identified using the NBS method (edge-level *p* < 0.001, component-level *p*_*FWE*_ < 0.05).

To investigate associations between ΔFC and symptom measures, we conducted canonical correlation analysis (CCA)—a multivariate statistical method that identifies correlations between two sets of variables [26]. One set of variables was ΔFC of all edges across the identified abnormal network; the other set of variables was clinical measures from each participant. For HC and AX, clinical measures including the Anxiety Sensitivity Index (ASI), Beck Anxiety Inventory (BAI), Beck Depression Inventory (BDI), and State Trait Anxiety Inventory-Trait form (STAI-T). For TENC and PTSD, the clinical measure was the Clinician-Administered PTSD Scale for DSM-5 (CAPS-5). We did not use the same clinical measures as in the HC and AX groups, since these measures were not collected in the TENC and PTSD groups. Nonparametric permutation tests (10000 times of shuffle) were employed to assess the statistical significance of the resulting canonical variables (see Supplementary Methods for details).

## Results

### Abnormal dynamic FC during extinction learning

**Fig. 1:**
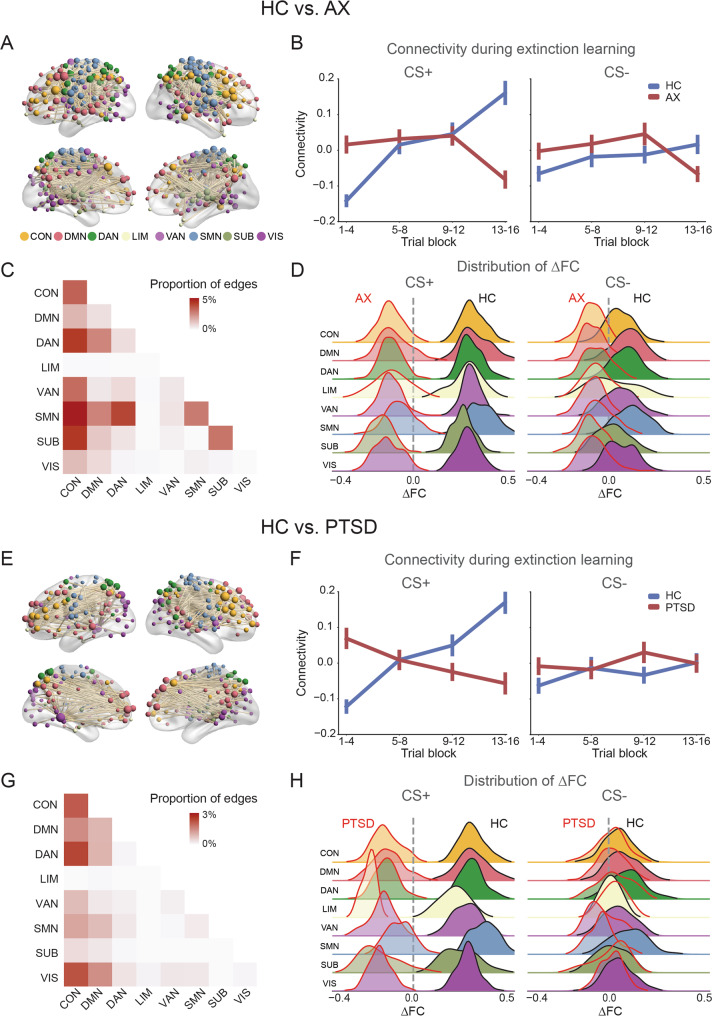
Abnormal dynamic connectivity in the AX and PTSD groups during extinction learning. **A** Network components showing significant differences in dynamic functional connectivity (FC) between HC and AX groups across multiple networks. Each sphere represents a brain region; the color of the sphere represents its network assignment, and the size of the sphere presents the weighted number of abnormal connections. **B** Mean FC of the identified network components during extinction learning for the CS+ and CS−. **C** The proportion of significantly impaired edges within or between the 8 subnetworks. A darkly shaded cell indicates that the connections of that network pair (indexed from *x*- and *y*-axis) were extensively impaired. **D** Distribution of mean connectivity change (ΔFC, comparing late (last 4 trials) minus early (first 4 trials) extinction learning) with each of the 8 subnetworks during CS+ or CS− processing. **E**–**H** panels are similar to panels **A**–**D**, but for HC vs. PTSD analyses. *AX* anxiety group, *HC* healthy controls, *PTSD* posttraumatic stress disorder.

As expected, we identified a network component showing widespread ΔFC differences in the AX group compared to the HC group (1369 edges, *p*_*FWE*_ < 0.001, Fig. 1A). During CS + processing, the mean FC of the HC group gradually increased from early to late extinction learning, while the mean FC pattern of the AX group decreased at the end of extinction learning (Fig. 1B). The identified component showed larger change of differential connectivity (difference between [CS+–CS−] at time-block 4 and [CS +–CS−] at time-block 1) in the HC than the AX group (*t*_*166*_ = 3.62, *p* < 0.001). The identified component did not show significant group differences during conditioning or extinction memory recall (see Supplementary Results, and Supplementary Fig. S1). We also constructed static FC—a single FC matrix for all CS+ trials. However, there was no significant statistical FC difference for HC vs. AX (CS + : *p*_*FWE*_ = 0.25), suggesting the importance of considering the extinction learning as a dynamic process.

We subsequently investigated to which specific brain networks do the identified abnormal between-group connections belong. We assigned regions to one of 8 pre-identified canonical networks involved in cognitive, attention, control, and sensory processing [27]. We calculated the proportion of significant edges among all edges within or between these networks. The abnormal edges in the AX group predominantly involved connections between the frontoparietal control (CON) and other networks, as well as between the dorsal/ventral attention (DAN/VAN) and somatomotor network (SMN), and these abnormalities were only observed during the processing of the CS+ and not the CS− (Fig. 1C, D).

We conducted the same analyses discussed above for the PTSD group. We first examined the abnormal network identified in AX, found that the PTSD group also showed lower extinction-induced ΔFC than the HC in this network (*t*_*156*_ = −4.46, *p* < 0.001). We then compared the PTSD group to HC group using whole-brain NBS procedure, which revealed a significant component (590 edges, *p*_*FWE*_ = 0.007, Fig. 1E). For the HC group, this component showed similar patterns to those identified in the HC vs. AX analyses, i.e., a gradual increase in FC from early to late extinction learning during CS + processing. In contrast, for the PTSD group, the mean FC pattern decreased across the extinction learning phase during the CS + processing, a pattern not observed during CS- processing (Fig. 1F). The identified component showed larger change of differential connectivity in the HC than the PTSD group (*t*_*156*_ = 4.95, *p* < 0.001). The abnormal edges in the PTSD group were predominantly noted within and between the CON, DMN, DAN/VAN, and visual networks; all of which were specific to the CS + and not to the CS− (Fig. 1G, H).

**Fig. 2:**
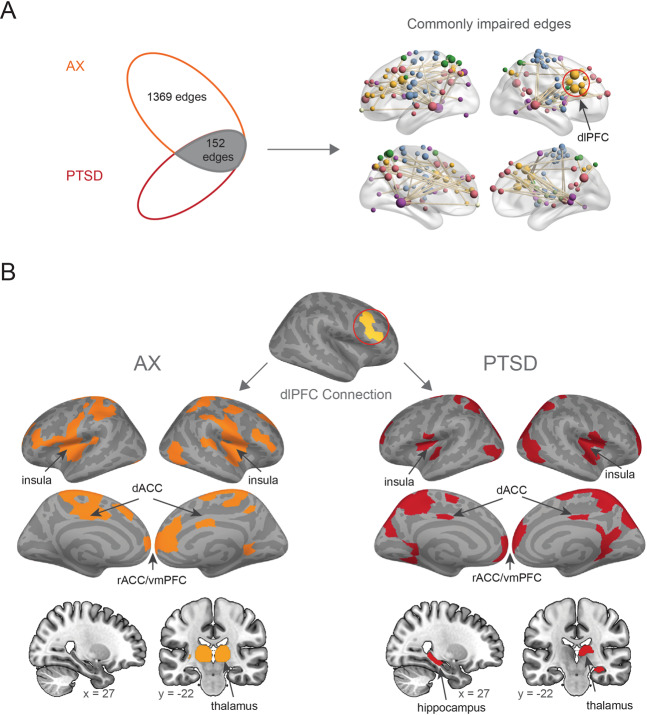
Shared and distinct impaired edges of the AX and PTSD groups during extinction learning. **A** There were 152 edges that were impaired in both AX and PTSD groups. Red circle highlights the dorsolateral prefrontal cortex (dlPFC) as an important hub with substantial overlapping impairments across the two disorders. **B** Separate display of the impaired FC between the dlPFC and the rest of the brain. Note substantial impairments in FC between dlPFC and key nodes of the ‘fear network’. *dACC* dorsal anterior cingulate cortex, *vmPFC* ventromedial prefrontal cortex, *rACC* rostral anterior cingulate cortex.

We next examined the extent of overlapping abnormalities in the ΔFC across the AX and PTSD groups. The intersection of the two identified components (HC vs. AX and HC vs. PTSD) was calculated. This analysis revealed 152 connections that were commonly impaired in the patient groups (Fig. 2A), largely involving the CON, DMN, and SMN (Fig. 2B). The dorsolateral prefrontal cortex (dlPFC) was one of the regions that formed the largest number of commonly impaired connections (Supplementary Fig. S2). Based on this result, we specifically examined which regions formed abnormal connections with the dlPFC in the two groups. Interestingly, the predominant impairments in the connections with the dlPFC across both groups were observed in regions composing the traditional “fear network”, including dACC, rostral ACC (rACC)/vmPFC and insula. One specific notable difference was that the PTSD group had an impaired dlPFC-hippocampus connection that was not observed in the AX group, whereas the AX group had an extensive dlPFC-thalamic impairment (Fig. 2B).

### Associations between extinction-induced FC and neural signals during extinction memory recall

**Fig. 3:**
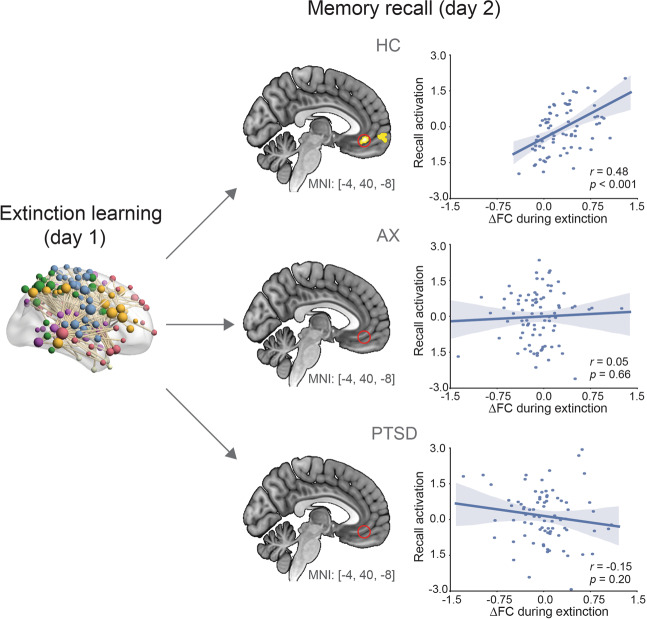
Change in functional connectivity during extinction learning predicts brain activation during extinction memory recall. The correlation between extinction-induced connectivity change and vmPFC activation during memory recall is significant within the HC, but not within the AX or PTSD group. Red circles in the AX and PTSD highlight the absence of any correlations from the same vmPFC location observed to be correlated within the HC. Scatter plots show correlations between change in FC and beta weights extracted from the vmPFC for each of the three groups.

Next, we examined whether ΔFC was associated with brain activation during extinction memory recall. For the network identified with HC vs. AX, the vmPFC was the only region that showed significant cross-phase correlation (Fig. 3). Specifically, the extinction-induced ΔFC (mean value of the identified component in Fig. 1A) positively related with the vmPFC activation at recall in the HC group (peak MNI_x,y,z_ = [−4,40,−8], *r* = 0.48, *p* < 0.001), but not in the AX group (*r* = 0.05, *p* = 0.45) or the PTSD group (*r* = −0.15, *p* = 0.20). Steiger’s Z test confirmed that the HC group showed significantly higher correlation than the other two groups (AX: Δ*r* = 0.43, *Z* = 2.93, *p* = 0.003; PTSD: Δ*r* = 0.63, *Z* = 4.07, *p* < 0.001). A significantly positive correlation between ΔFC and vmPFC activation was also observed in the HC group using the network identified with HC vs. PTSD (Supplementary Fig. S3).

**Fig. 4:**
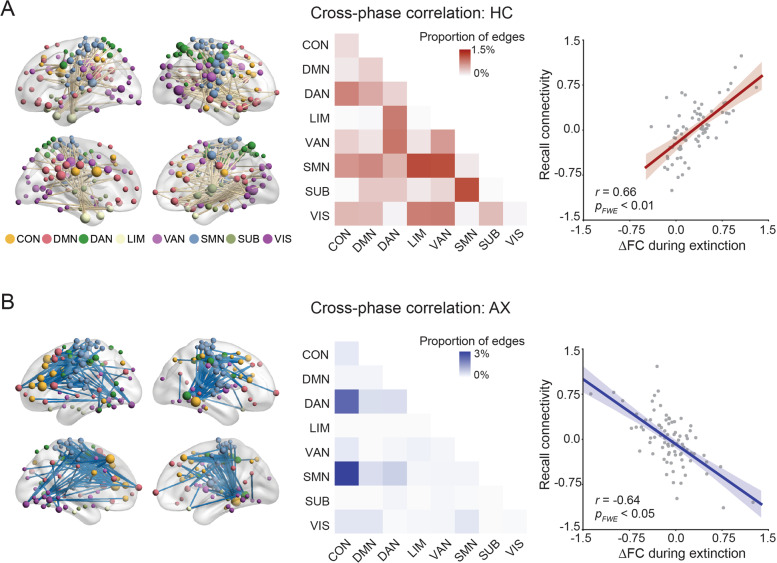
Change in functional connectivity during extinction learning correlates with functional connectivity during memory recall test. **A** The FC change positively correlated with FC during extinction memory recall within the HC group. **B** The FC change negatively correlated with connectivity during memory recall within the AX group.

We next examined the correlation between ΔFC and connectivity during extinction memory recall. For the HC group, there was a network component that exhibited positive cross-phase correlation (419 connections, *p*_*FWE*_ = 0.015, Fig. 4A). The network implicated distributed connections between networks, especially between the SMN and DMN. In contrast, the AX group exhibited a negative correlation between ΔFC and FC (344 connections, *p*_*FWE*_ = 0.020, Fig. 4B). The network mainly involved connections between the CON and other networks, especially the SMN and VAN.

### Comparing PTSD with TENC

The above analyses for the PTSD cohort involved a HC group as the control group. This enabled comparisons of the results between the AX and PTSD cohorts. Given that prior studies commonly use a trauma-exposed non-PTSD controls (TENC) group as a control group, we conducted additional FC analyses comparing the PTSD to TENC. These additional analyses mostly replicated the results of HC vs. PTSD analyses (see Supplementary Results for details); revealing a significant network component that mainly involved connections between the DMN with other networks (Supplementary Figs. S4 and S5). The TENC group showed increased FC while the PTSD showed decreased FC, from early to late extinction learning—changes that were only observed to the CS+.

### ΔFC associations with clinical measures across all participants

**Fig. 5:**
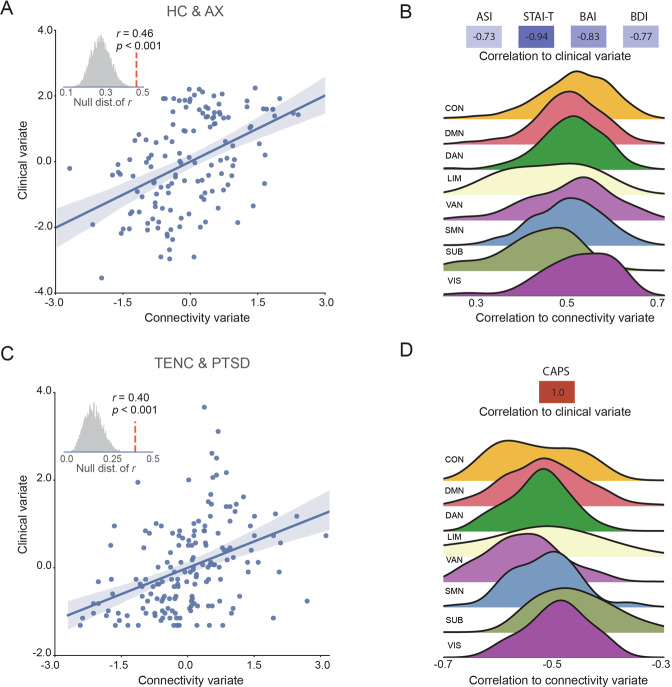
The canonical correlation analysis between connectivity change during extinction learning and clinical measures. **A** The correlation between connectivity variate and clinical variate is significant for the HC and AX cohort. **B** The canonical loadings of the clinical measures (top) and connectivity changes with each of the 8 subnetworks (bottom). **C**, **D** Same as in panels **A** and **B**, but for the TENC and PTSD cohort.

CCA identified a significant canonical variate linking ΔFC to the clinical measures (i.e., ASI, STAI-T, BAI and BDI) in the HC and AX groups (*r* = 0.46, permutation test *p* = 0.007, Fig. 5A). We further conducted a 5-fold cross-validation procedure, which repeatedly trained the CCA model on 80% of the data, tested the trained model on the remaining 20% data (see Supplementary Methods for details). This procedure resulted in a significant correlation between the predicted and observed measures (*r* = 0.40, permutation test *p* = 0.003), supporting the robustness of the identified canonical variate. We then calculated the canonical loadings to examine how the individual clinical measure and connectivity contributed to the canonical variate (Fig. 5B). The analysis revealed that all 4 clinical measures negatively contributed to the clinical score, while extinction-induced ΔFC across the 8 networks positively correlated with the connectivity variate.

Similar results were observed in the TENC and PTSD groups. Specifically, there was a significant canonical variate linking ΔFC to the symptom measure (CAPS-5) in the TENC and PTSD groups (*r* = 0.40, permutation test *p* < 0.001, Fig. 5C). The 5-fold cross-validation procedure further confirmed the identified link (*r* = 0.36, permutation test *p* < 0.001). The canonical loadings indicated that the symptom measure positively contributed to the clinical variate, while ΔFC negatively correlated with the connectivity variate (Fig. 5C). Together, these results suggest that the lower magnitude of change in functional connectivity during extinction learning was associated with higher levels of fear- and anxiety-related clinical measures.

## Discussion

We studied the dynamic changes of large-scale FC across extinction learning in healthy controls (HC) and patients diagnosed with anxiety disorders (AX) or PTSD. From early to late extinction learning, HC exhibited an increase in FC that was specific to the conditioned cue. The AX and PTSD groups exhibited widespread FC impairments during extinction learning. The relative FC reductions in patient groups were predominantly in the interactions between the default mode network (DMN), frontoparietal control network (CON), somatomotor network (SMN), and attention networks (DAN/VAN). The extinction-induced FC changes were predictive of vmPFC activation during extinction recall only in the HC group. FC changes during extinction learning positively and negatively correlated with FC during recall in the HC and the AX groups, respectively. Finally, extinction-induced FC was associated with subjective measures of fear- and anxiety-related clinical metrics.

Animal literature shows that learning-induced plasticity can change both neural activity and neuronal synchronization [28]. Neural activity change is presumably reflected in changes in regional activation, whereas neuronal synchronization between distributed regions may better be characterized by functional connectivity [29]. Prior fMRI literature on fear conditioning and extinction has focused on localized brain activation. Abnormal activations during extinction learning and/or fear recall/renewal were reported in populations with anxiety disorders and PTSD, especially within the ‘fear network’ [19, 30–34]. Structural abnormalities beyond the ‘fear network’ were robustly observed in PTSD [35]. In this study, we shifted our analytic strategy to pay more attention on the dynamic functional connectivity during the experiment. Our results extend prior studies by showing that interactions between brain regions extensively beyond the ‘fear network’ were altered in PTSD and AX groups during extinction learning. The observed cross-phase correlations are in line with animal studies showing that neural changes during learning are relevant to the retrieval [36, 37]. We also examined the association between the change of connectivity and activation within the extinction learning. This analysis did not show significant association in the HC or PTSD group, but revealed significant negative correlation between connectivity and activation (especially in regions like insula and dACC) in the AX group (Supplementary Figure S6). These results suggest that the activation and connectivity contain distinct information, examining both activation and connectivity can provide complementary information for the understanding of pathophysiology [38].

Prior fear extinction studies have focused predominantly on neural circuits associated with expression and inhibition of conditioned threat responses. The conscious awareness of fear, and the feeling of no longer being afraid, would require multiple cognitive processes, including perception, attention, conscious awareness, and memory construction. These multi-level processes are likely to require coordinated interactions between distributed brain regions [39]. A recently proposed “two-system” framework view of fear suggests that higher-order association cortices would be needed to generate and regulate the feeling of fear [11]. In support of this view, our recent study on HC showed the engagement of distributed brain regions during extinction learning [14]. These brain regions were part of many networks including the DMN, CON, DAN/VAN and SMN. These networks are extensively involved in conscious awareness, attentional control, memory encoding, perception and motor control [27, 39]. Furthermore, previous studies found that activations across multiple brain systems are engaged in fear-related processing [12, 13]. Dysfunction within and between the DMN, CON, and SMN were extensively reported in resting-state and task-based fMRI studies investigating psychiatric disorders [40, 41]. The abnormal FC across the above noted networks in patient groups suggests inadequate encoding and/or consolidating of information related to perception and conscious awareness of threat-inducing stimuli. These impairments in higher-order processing could thereafter lead to imbalance in the maintenance of fear and emotional homeostasis.

The distinct dysfunction in some networks and the partial overlap in some others across the AX and PTSD groups suggest both divergent and shared mechanistic impairments pertinent to fear inhibition across these psychopathologies. While there was an overlap in the FC dysfunction across multiple brain regions between the patient groups, the dlPFC—a region known for its contribution to cognitive control and regulation [42–44], appeared to be highly relevant. The dlPFC was activated when subjects were instructed to regulate their conditioned fear [42, 43]. Importantly, transcranial magnetic stimulation-based neuromodulation targeting the dlPFC during [45] or after [46] extinction learning enhanced extinction memory recall in HC, demonstrating the important role of dlPFC during extinction learning. The dysfunctional connections between the dlPFC across the two patient groups were mostly associated with the dACC, insular cortex, and vmPFC. These brain regions are key nodes of the ‘fear network’, and have been shown to be dysfunctional across anxiety disorders and PTSD. A notable distinction is that dlPFC-hippocampus FC was impaired in the PTSD group only, whereas insular cortex-dlPFC dysfunction in FC was much more pronounced in the AX group. These data are consistent with the idea that anxiety disorders are more often associated with imbalance in the perception and regulation of internal states (mostly related to insular dysfunction) [47], whereas PTSD is a disorder that is more often associated with failure to contextualize certain experiences associated with the trauma—a process that is heavily reliant on the hippocampus (see [30, 48] for comprehensive review).

We observed a positive correlation between extinction-induced ΔFC (predominantly involved CON, DMN, DAN and SMN) and vmPFC activation during memory recall only in the HC group. The vmPFC plays a key role in inhibiting threat response [3, 6, 20, 49]. Abnormal vmPFC activation during the retrieval of extinction memory was observed in individuals with PTSD or anxiety disorders [16, 19, 31], which may relate to the deficits in extinction memory recall. The extinction-induced ΔFC was also differentially associated with FC during extinction memory test for the HC and patient groups. Specifically, ΔFC positively correlated with distributed connections in the HC group, while negatively correlated with a network component dominated by connections between CON and SMN in the AX group, suggesting that psychopathology impacts the encoding of extinction memory and thus leads to deficits in its retrieval. This inefficient encoding of extinction learning might be related to, or caused by, an initially higher degree of state or trait anxiety. This ‘higher load of emotionality’ would therefore interfere with the cognitive processes required to learn that a given cue is no longer predictive of danger. Indeed, we observed a significant association between extinction-induced FC change and subjective reports of anxiety symptoms (including ASI, STAI-T and BAI); the higher the anxiety and trauma-related symptoms (CAPS-5), the lower the magnitude of FC observed during extinction learning—especially connections with CON and VAN. These results provide support for the two-system concept proposed by LeDoux and Pine. That is, conscious feelings of fear and anxiety require higher-order brain processing; impairments of these systems lead to lesser cognitive attention and sensory processing of highly relevant information. Less cognitive processing then leads to imbalance in fear perception and its homeostasis.

We specifically focused our analyses on the connectivity during CS+ processing since our previous study on healthy controls showed that connectivity during CS+ processing increased from early to late extinction learning, and predicted the magnitude of extinction memory [14]. The identified network components also showed significantly higher change of differential connectivity (CS + vs. CS−) from early to late extinction learning in the HC group than the AX/PTSD group, suggesting learning-induced plasticity that is specific to the conditioned cue. In addition to the analytic approach discussed above, we further conducted generalized psychophysiological interactions (gPPI) analysis [50] to investigate the change of differential connectivity at whole-brain level. We identified abnormal change of differential connectivity in the AX (Supplementary Fig. S7) but not PTSD group. The lack of significant interaction difference for HC vs. PTSD, as discussed in previous study [31], might be due to the overgeneralization of PTSD patients across cues (CS+ and CS−) [51], which could undermine detection of differential effects. An alternative explanation to the negative finding in the PTSD cohort is that gPPI and beta-series correlation methods have relatively low sensitivity in detecting subtle difference between conditions [52]. This is especially the case in event-related design with small number of trials, which may lower the power in detecting complex stimulus-by-group interactions.

We observed abnormal connections extensively involved CON and DMN in the PTSD group when comparing it with either the non-exposed controls or trauma-exposed controls. This result suggests that the connectivity alterations in the PTSD group are not merely a result of the traumatic exposure. There is evidence showing that traumatic exposure per se may alter activation and functional connectivity [17, 53, 54]. Therefore, it is important for future studies to have three-group design (i.e., include both non-exposed, trauma-exposed controls, and PTSD) to further understand effects of trauma exposure and PTSD mechanisms (see [55] for detailed discussion). In this study, the anxiety group was composed by subjects diagnosed with different types of anxiety disorders. The small sample size of each specific anxiety type prevented us from conducting a subgroup analysis to explore the contribution of each specific diagnosis to the detected abnormal functional connectivity. It would be interesting for future studies to investigate whether different anxiety disorders exhibit similar patterns of abnormal connectivity with a larger sample size from each anxiety type.

The dynamic nature of extinction learning-induced neural plasticity is overlooked to some degree in prior studies as many neuroimaging studies average functional brain activation across extinction learning. However, a few prior studies suggested the dynamic changes of activation during the fear conditioning and extinction paradigm in PTSD and healthy participants [19, 31]. Specifically, activation patterns and case-control differences clearly differed when extinction and extinction recall phases were further divided into “early” and “late” stages. Our study extends these studies by directly examining the dynamic change of functional connectivity between groups with more refined temporal resolution. Together, these studies provide strong support to the idea that a critical focus on the dynamic nature of learning during an experiment is needed and essential. We demonstrated that individuals with fear- and anxiety-related disorders exhibited widespread impairment in connectivity patterns- captured when considering time (number of trials during learning)- compared with controls, particularly involving the default mode network, frontoparietal control network, and somatomotor network. The modulation of functional connectivity during extinction learning was associated with clinical measures and impacted neural signals during extinction memory recall the next day. Our results suggest that distributed network interactions may contribute to the deficits of extinction memory recall in fear- and anxiety-related disorders.

## Supplementary information

Supplemental material
