# Supplementary material for: Abnormal dynamic functional connectivity during fear extinction learning in PTSD and anxiety disorders
Source: Mol Psychiatry. 2022 Feb 10;27(4):2216–24. doi: 10.1038/s41380-022-01462-5 (PMC9126814; doi:10.1038/s41380-022-01462-5)
Supplement: Supplementary file 1 — Supplemental material [file 41380_2022_1462_MOESM1_ESM.docx]

**Supplemental Material**

**Abnormal dynamic functional connectivity during fear extinction learning in PTSD and anxiety disorders**

Zhenfu Wen, Jeehye Seo, Edward F. Pace-Schott, Mohammed R. Milad

**Methods**

**Participants**

Participants were included in the study if they met the following inclusion criteria: 18-65 years old, proficient in English, right-handed, and normal or corrected-to-normal vision. The exclusion criteria included: history of seizures or significant head trauma, current substance abuse or dependence, metal implants, pregnancy, breastfeeding, or positive urine toxicology screen for drugs of abuse. The healthy control (HC, N = 77) group included individuals without a psychiatric disorder. The anxiety group (N = 91) included subjects diagnosed with the following anxiety disorders: generalized anxiety disorder (N = 27), social anxiety disorder (N = 27), specific phobia (N = 20), and panic disorder (N = 17). The posttraumatic stress disorder (PTSD, N = 81) group and the trauma-exposed non-PTSD controls (TENC, N = 89) included participants who had experienced at least one DSM-IV criterion-A traumatic event (see **Supplemental Table S1** for sample demographics). The Clinician Administered PTSD Scale (CAPS) was administered to evaluate PTSD symptom severity. All procedures were approved by the Partners HealthCare Institute Review Board of the Massachusetts General Hospital, Harvard Medical School. All participants provided written informed consent before they participated the study.

**Experimental procedure**

The 2-day fear conditioning and extinction paradigm consisted of fear conditioning (day 1), fear extinction learning (day 1), and extinction memory test (day 2). Before the experiment, participants were instructed to select the level of electric stimulation to be used throughout the experiment, so that the stimulation level was highly annoying but non-painful. Electrical stimulation was delivered through electrodes that were attached to the index finger and middle finger of the participant’s dominant (right) hand. The paradigm then started with a habituation stage during which all paradigm images were presented to the participant, without any electrical stimulation. Following the habituation, fear conditioning occurred in one context (e.g., the ‘office’ picture) where two of the colored lamps (e.g., blue and red, CS+) were partially reinforced (62.5% reinforcement rate) with a mild electric shock (500 ms, occurring at the offset of CS presentation) and the third colored lamp (e.g., yellow) was never paired with the shock (CS-). Fear conditioning consisted of a total of 32 trials (8 presentations for each of the two CS+s and 16 presentations of CS-). After conditioning, extinction learning occurred in a different context (e.g., the library), where one of the CS+ was presented 16 times without any shocks (CS+ extinguished; CS+E) intermixed with 16 presentations of CS-. On day 2, extinction memory retention was tested in the context of extinction learning (e.g., the ‘library’ picture), where the three colored lamps were presented (8 presentations of the extinguished CS+E, 8 presentations of the unextinguished CS+ (CS+U) along with 16 presentations of CS-). The order of stimulus presentation was pseudo-random for all stages of the paradigm. The trial structure was identical across different phases. Each trial started with a blank screen lasting 12-18 s (mean: 15 s), followed by a picture of a room (either a library or an office) with an unlit lamp for 3 s (context) after which the lamp turned on to blue, red or yellow and lasted for 6 s (conditioned stimulus, CS).

**MRI acquisition and preprocessing**

The neuroimaging data were acquired in a Trio 3.0 Tesla whole-body MRI scanner (Siemens Medical Systems, Iselin, NJ) using a 32-channel head coil. Anatomical images were acquired for image registration. Functional data were acquired using a T2*-weighted echo-planar imaging (EPI) pulse sequence (TR: 2.56 s, TE: 30 ms, voxel size: 3 × 3 × 3 mm). Preprocessing was performed using fMRIPrep 20.0.2 with default settings. Functional images were corrected for slice timing, realigned, co-registered with the structural image, normalized into the Montreal Neurological Institute (MNI) space, and smoothed with an 8-mm full width half-maximum Gaussian kernel.

**Dynamic functional connectivity estimation**

We focused on the dynamics of whole-brain functional connectivity (FC) during extinction learning. As in our previous study (1), we estimated the single-trial FC using a jackknife procedure (2,3). The procedure included the following steps. First, we estimated brain activation for each single trial (beta image) using the least-squares-all-based generalized linear model (4). The model included a regressor for each of the CS presentation (32 regressors in total), a regressor for the context presentation. Each regressor was modeled by convolving the onset of CS with the canonical hemodynamic response function. Other regressors including the motion parameters, high-pass temporal filtering (128 s) terms, polynomial drift were included in the model. A first-order autoregressive (AR) model was used to account the temporal structure of the noise. Second, we extracted regional beta values using a whole-brain parcellation consisting of 400 cortical regions (5) and 32 subcortical regions (6), so that we obtained a 432-dimensional beta vector for each trial. Third, we combined the beta series correlations method (7) and the jackknife correlation method to estimate FC (1–3). Specifically, we left out the beta vector of a specific trial (from the 32 trials), then calculated a pair-wise regional Pearson’s correlation based on the 31 beta vectors, which resulted in a 432 × 432 symmetric matrix for each trial. This procedure was repeated for each of the 32 trials to obtain 32 connectivity matrices. The elements of the matrices were then z-scored across trials. Finally, we reordered these 32 matrices of all trials according to CS type (i.e., CS+ and CS-) and presentation time (i.e., from the first to the last CS trial) during the experiment. To increase signal-to-noise ratio, we divided the trials of each CS type into 4 time-blocks (from 1 to 4, representing early to late extinction learning), averaged the 4 trials within each time-block. After the procedure, we obtained 4 FC matrixes during CS+ processing, 4 FC matrixes during CS- processing, for each participant in the extinction learning phase. Our prior study in HC showed that the largest and most significant change in FC occurred between blocks 1 (first 4 trials) and block 4 (last 4 trials of extinction) during CS+ processing. Therefore, we focused our analyses in this study on the difference between these two blocks (ΔFC, defined as FC in time-block 4 minus FC in time-block 1, during CS+ processing).

We also estimated dynamic FC during fear conditioning and extinction memory recall phases. For fear conditioning, trials of each of the two CS+s were divided into 2 time-blocks (4 trials per time-block) and averaged within time-block. The FC matrices were further averaged across CS+s, so that we got two matrices for CS+ processing, one for early conditioning, the other for late conditioning. For extinction memory test, we specifically focused on the early memory recall—the first four trials of each of the CS type (CS+E, CS+U, and CS-), to minimize the confound introduced by additional extinction learning during later trials of memory recall. We got a matrix for CS+E, CS+U, and CS-, respectively.

**The NBS analyses**

We used the Network-Based Statistic (NBS) procedure to identify network component that showed significant difference between groups. The NBS analysis included the following steps: 1) we compared each element/edge of the upper-matrix of ΔFC (a total of 93096 edges) between the two groups (HC vs. AX, or HC vs. PTSD) using two-sample t-test, and threshold edges with a *p* < 0.001 into a set of suprathreshold edges; 2) we identified connected components within the suprathreshold edges, and calculated the sizes of these components; 3) we shuffled the labels of participants, and then conducted steps 1 and 2 to estimate the maximal size of connected component. A null distribution of the size of connected component were obtained by repeating the procedure 10000 times; 4) we then counted the number of values within the null distribution that were larger than the real component size, then divided it by 10000 to obtain the *p* value. A *p* value smaller than 0.05 indicated family-wise error corrected significance of the identified network component. Before the NBS analysis, the age and sex regressors were regressed out from each element of the ΔFC matrix using linear regression model across participants. The residuals of the linear regression mode were entered into the NBS procedure.

**Canonical correlation analyses**

To investigate associations between ΔFC and symptom measures, we conducted canonical correlation analysis (CCA). CCA is a multivariate statistical method that identifies linear combinations of two sets of variables that maximally correlate with each other (8). We separately conducted CCA on two cohorts, with one cohort comprised of the HC and AX groups, the other cohort comprised of the PTSD and TENC groups. We made this choice because different clinical measures were collected for the two cohorts. For HC and AX, clinical measures including the Anxiety Sensitivity Index (ASI), Beck Anxiety Inventory (BAI), Beck Depression Inventory (BDI), and State Trait Anxiety Inventory-Trait form (STAI-T). For TENC and PTSD, the clinical measure was the Clinician-Administered PTSD Scale (CAPS).

The CCA included the following steps. 1) We extracted ΔFC value for each connection of the identified network component (HC vs. AX, or TENC vs. PTSD), regressed out the age and sex effects and retained the residuals. 2) We then averaged connections within or between the 8 canonical networks, which lead to 36 values (8 values for within networks, 28 values for between every two networks); we further conducted principal component analysis (PCA), retained 5 components (all components accounted for more than 4% of variance) as the connectivity measures. We conducted the above procedures to summarize the high-dimensional edgewise ΔFC values (1369 values for HC and AX, 230 values for TENC and PTSD) into lower dimension, since large number of variables likely leads to overfitting in CCA (8). 3) We then fit the CCA model to assess the correlation between connectivity measures and clinic measures. Conceptually, CCA seeks linear combinations of variables of both sets (connectivity measures and clinic measures), such that the correlation between the two combined variables (the connectivity variate and the clinic variate) was maximized (see (8) for details). 4) We assessed statistical significance of the obtained CCA variates (correlations) using permutation test. Specifically, we randomly shuffled the clinic measures across participants, and ran the CCA model to get CCA variates. The shuffling procedure was repeated for 10000 times, so that we got the null distribution of CCA variates. The *p*-value was calculated by dividing the number of values in the null distribution that were larger than the non-shuffled CCA variates by 10000. 5) We then calculated the canonical loadings to examine how the individual clinical measure and connectivity contributed to the canonical variate. For the connectivity variate, we calculated the correlation between it and each of the original ΔFC value. For the clinic variate, we calculated the correlation between it and each of the original clinic measure. A higher absolute correlation value suggests the original variable contributed more in deriving the canonical variate.

We further conducted a 5-fold cross-validation analysis to assess the significance of CCA. In this analysis, we randomly divided the participants into 5 folds, kept data from 4 folds as training set, leaving data from the other fold as testing set. We train a CCA model only based on the training set, and applied the trained model to the testing set. Specifically, we took the combination weights learned from the training set, and multiplied those into the left-out testing set to get the predicted connectivity variate and clinic variate. This procedure was repeated 5 times, each time with a different fold of data as testing set. We then calculated the correlation between the predicted connectivity variate and clinic variate. We used permutation test to assess the significance of the cross-validation analysis. We shuffled the clinic measures across participants (10000 times) and re-do the 5-fold cross-validation analysis to obtain null distribution of the correlation value between the predicted connectivity variate and clinic variate.

**Results**

**The abnormal network component during fear conditioning and memory recall**

For the identified abnormal network components during extinction learning (HC vs. AX, or HC vs. PTSD), we tracked their mean functional connectivity values during fear conditioning and extinction memory recall (**Supplemental Figure S1**). We separately examined early and late conditioning, and early extinction memory recall. For the network identified with HC vs. AX, a CS-type (CS+ or CS-) × Group (HC or AX) mixed-ANOVA revealed a main effect of CS-type (*F*(1,152) = 4.6, *p* = 0.03) in early fear conditioning, but it did not survive the multiple comparison correction. No other effect was significant (*p*s > 0.1). For the network identified with HC vs. PTSD, we did not observe any significant results in either early/late fear conditioning or early extinction memory recall (*p*s > 0.1).

**Commonly impaired connections with each brain region**

The 152 connections that were commonly impaired in the AX and PTSD groups involved 120 brain regions. For each of the 120 regions, we calculated the number of commonly impaired edges that connected with it as nodal weight. A region with larger nodal weight suggests that this region formed extensive FC impairments during extinction learning in both the AX and PTSD groups. We ranked the regions based on their nodal weights, which revealed that the dorsolateral prefrontal cortex (dlPFC) was one of the regions with the largest nodal weight. Specifically, among the 5 regions with the largest nodal weights, 3 (2 from right brain, 1 from left brain) of them belong to the dlPFC; among the 10 regions with the largest nodal weights, 5 (4 from right brain, 1 from left brain) of them belong to the dlPFC. Considering the important role of the dlPFC in cognitive regulation, we specifically focused on this region. In the main manuscript, the dlPFC cluster was defined by combining the 4 regions in the right hemisphere since they formed a larger cluster than the left dlPFC. These results are shown in supplemental figure S2.

**Comparing PTSD with TENC**

We compared the extinction-induced ΔFC between PTSD and TENC using the whole-brain NBS method. This analysis revealed a significant network component comparing 230 connections (*p_FWE_*<0.05, **Supplemental Figure S4A**), that mainly involved connections between the default mode/frontoparietal control network with other networks (**Supplemental Figure S4C**). During CS+ processing, the TENC group showed increased FC while the PTSD showed decreased FC, from early to late extinction learning- changes that were only observed to the CS+ (**Supplemental Figure S4B, S4C**). We further conducted cross-phase correlation analyses based on the identified network component. We did not observe a significant correlation between ΔFC and brain activation during extinction memory recall in both groups. However, in the TENC group, there was a significant network component during extinction memory recall that showed positive correlation with ΔFC (*p_FWE_*<0.05, **Supplemental Figure S5**). This component mainly involved connections between ventral/dorsal attention and frontoparietal control networks.

**Association between connectivity and activation change within extinction learning**

In addition to the cross-phase correlation analysis, we examined the association between the change of connectivity and activation within the extinction learning. In this multiple regression analysis, the independent variable was the mean ΔFC across the identified components, the dependent variable was the brain activation change during extinction learning (difference between differential activation [CS+ – CS-] at time-block 4 and [CS+ – CS-] at time-block 1). Significant associations were identified at a voxel-level *p*<0.001 and a cluster-level *p_FWE_*<0.05. This analysis did not show significant association in the HC or PTSD group, but revealed significant negative correlation between connectivity and activation in the AX group. The regions showed significant negative correlation largely involved insula and dACC (**Supplemental Figure S6**).

**Generalized Psychophysiological Interactions analysis**

We further conducted generalized psychophysiological interactions (gPPI) analysis (9) to investigate the change of differential connectivity (CS+ vs. CS-) from early to late extinction learning. Specifically, we divided the extinction learning phase into 4 time-blocks as in the main manuscript (4 trials of each CS type in a time-block), and estimated whole-brain gPPI connectivity at each time-block. We then used the network-based statistics (NBS) method (10) to identify network component showing group difference between differential connectivity ([CS+ – CS-]) at time-block 4 and [CS+ – CS-] at time-block 1. Significant network components were identified using a edge-level *p*<0.001 and a component-level *p_FWE_*<0.05. For HC vs. AX, the NBS method identified a network component showing larger change of the connectivity difference in the HC group than the AX group (282 edges, *p_FWE_*=0.025, **Supplemental Figure S7**). We did not observe significant difference between the HC and PTSD group (largest network component: 54 edges showing HC > PTSD, *p_FWE_*=0.28).

**Reference**

1. Wen Z, Chen ZS, Milad MR (2021): Fear extinction learning modulates large-scale brain connectivity. *NeuroImage* 238: 118261.

2. Richter CG, Thompson WH, Bosman CA, Fries P (2015): A jackknife approach to quantifying single-trial correlation between covariance-based metrics undefined on a single-trial basis. *NeuroImage* 114: 57–70.

3. Thompson WH, Richter CG, Plavén-Sigray P, Fransson P (2018): Simulations to benchmark time-varying connectivity methods for fMRI. *PLOS Computational Biology* 14: e1006196.

4. Mumford JA, Turner BO, Ashby FG, Poldrack RA (2012): Deconvolving BOLD activation in event-related designs for multivoxel pattern classification analyses. *NeuroImage* 59: 2636–2643.

5. Schaefer A, Kong R, Gordon EM, Laumann TO, Zuo X-N, Holmes AJ, *et al.* (2018): Local-Global Parcellation of the Human Cerebral Cortex from Intrinsic Functional Connectivity MRI. *Cereb Cortex* 28: 3095–3114.

6. Tian Y, Margulies DS, Breakspear M, Zalesky A (2020): Topographic organization of the human subcortex unveiled with functional connectivity gradients. *Nature Neuroscience* 1–12.

7. Rissman J, Gazzaley A, D’Esposito M (2004): Measuring functional connectivity during distinct stages of a cognitive task. *NeuroImage* 23: 752–763.

8. Wang H-T, Smallwood J, Mourao-Miranda J, Xia CH, Satterthwaite TD, Bassett DS, Bzdok D (2020): Finding the needle in a high-dimensional haystack: Canonical correlation analysis for neuroscientists. *NeuroImage* 216: 116745.

9. McLaren DG, Ries ML, Xu G, Johnson SC (2012): A generalized form of context-dependent psychophysiological interactions (gPPI): A comparison to standard approaches. *NeuroImage* 61: 1277–1286.

10. Zalesky A, Fornito A, Bullmore ET (2010): Network-based statistic: Identifying differences in brain networks. *NeuroImage* 53: 1197–1207.

**Supplementary Table S1.** Sample demographics.

|  | Healthy controls | Anxiety | Trauma-exposed non-PTSD | PTSD |
| --- | --- | --- | --- | --- |
| Number of subjects | 77 | 91 | 89 | 81 |
| Gender (Female/Male) | 54/23 | 59/32 | 48/41 | 63/18 |
| Age (mean ± SD) | 26.4 ± 7.4 | 30.4 ± 11.9 | 27.8 ± 10.3 | 28.4 ± 10.1 |
| ASI (mean ± SD) | 17.6 ± 10.2 | 26.9 ± 13.4 | nan | nan |
| BAI (mean ± SD) | 2 ± 2.8 | 20.0 ± 12.7 | nan | nan |
| BDI (mean ± SD) | 3.3 ± 3.4 | 14.7 ± 11.6 | nan | nan |
| STAI-T (mean ± SD) | 33.9 ± 7.6 | 50.7 ± 10.1 | nan | nan |
| CAPS (mean ± SD) | nan | nan | 10.2 ± 8.3 | 38.0 ± 14.1 |

Abbreviations: CAPS (Clinician Administered PTSD Scale), ASI (Anxiety Sensitivity Index), BAI (Beck Anxiety Inventory), BDI (Beck Depression Inventory), STAI-I (State Trait Anxiety Inventory-Trait form)


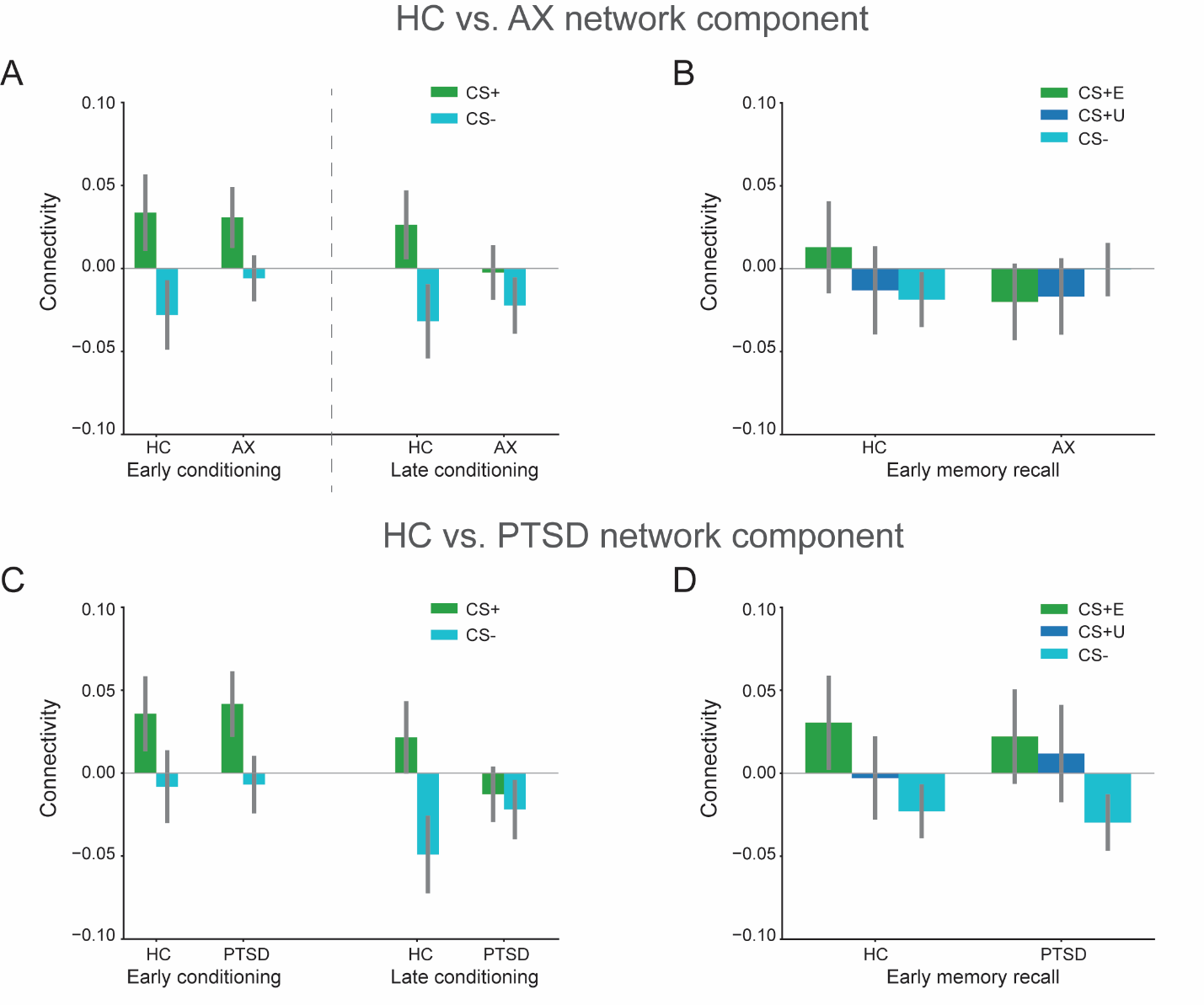


**Supplementary Figure S1.** The mean functional connectivity of the identified abnormal network component during fear conditioning and memory recall. For the network component identified with HC vs. AX, the mean functional connectivity during early and late conditioning **(A)** and during early memory recall test **(B).** For the network component identified with HC vs. PTSD, the mean functional connectivity during early and late conditioning is shown in **C** and the mean functional connectivity during early memory recall test is shown in **D**.


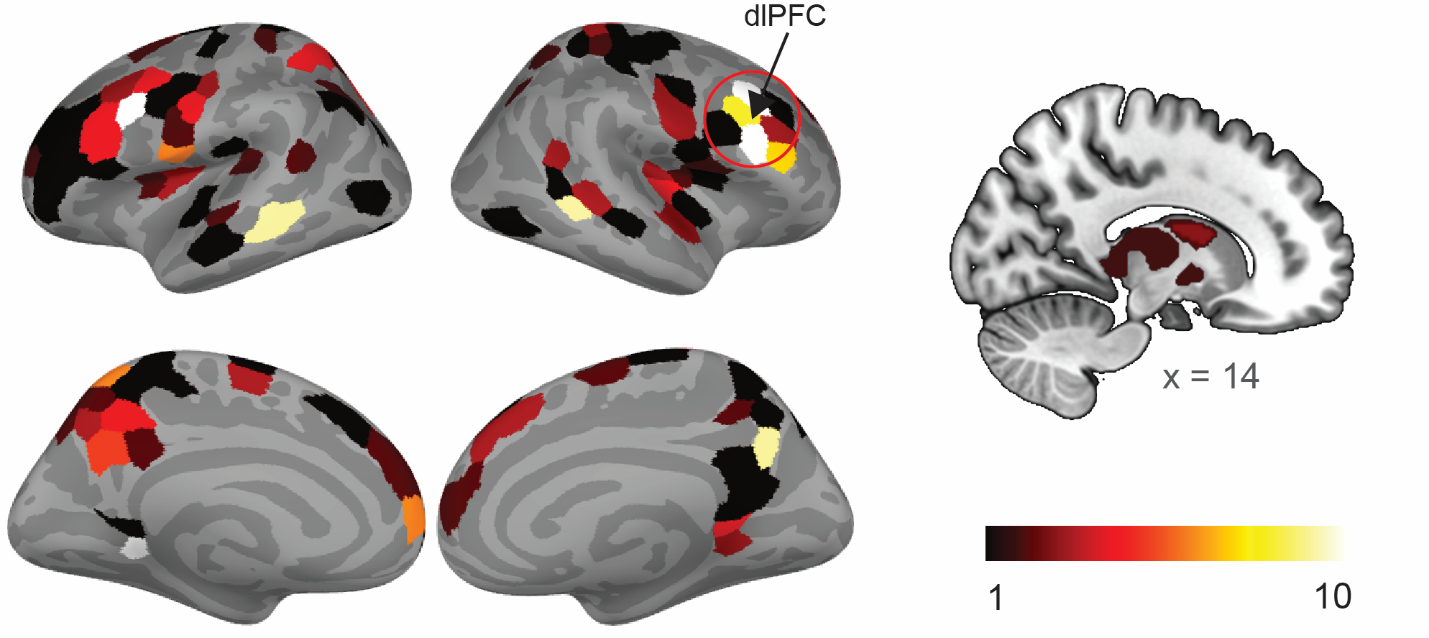


**Supplementary Figure S2.** Number of commonly impaired functional connectivity in anxiety and PTSD patients with each brain region during extinction learning. DLPFC: dorsolateral prefrontal cortex


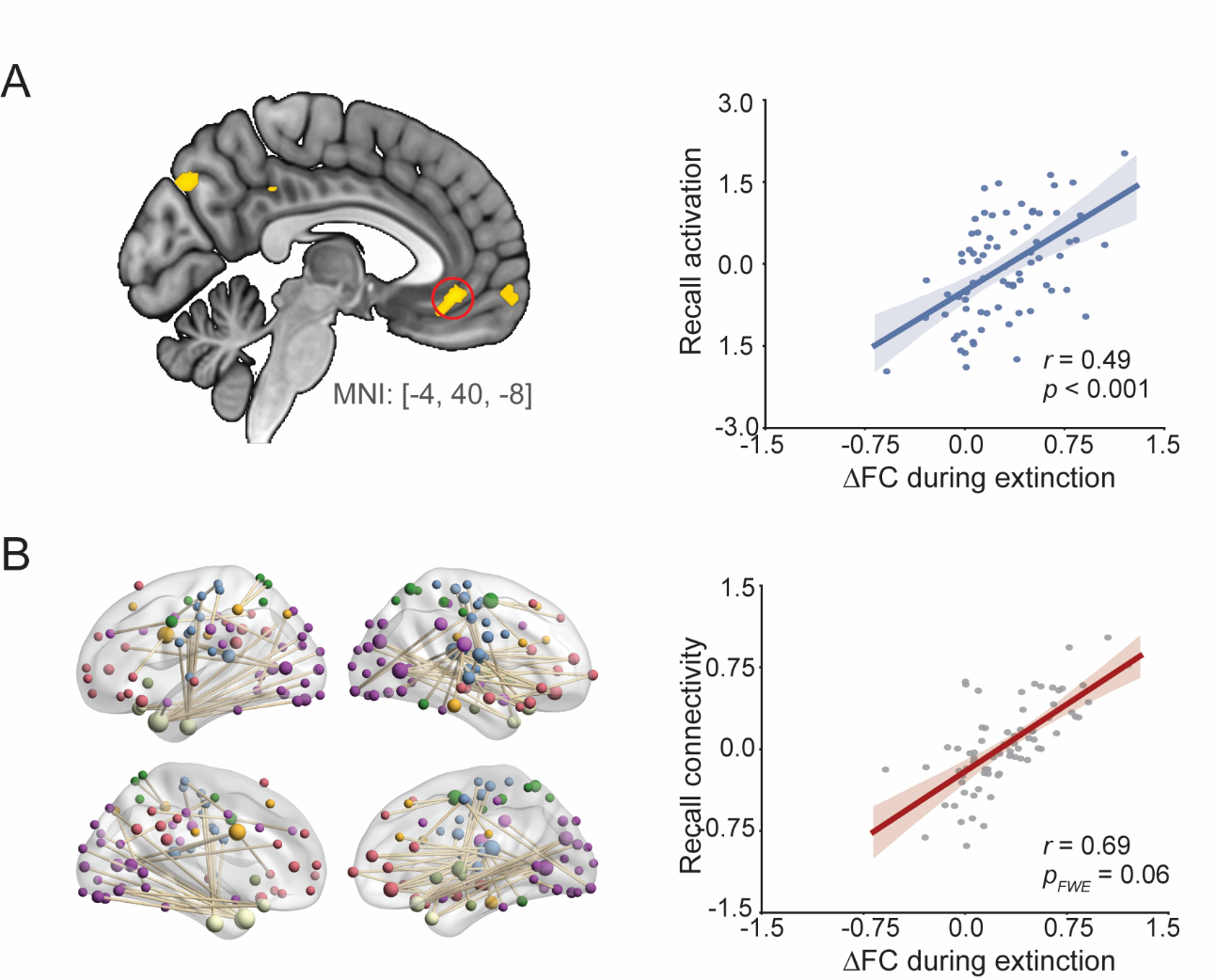


**Supplementary Figure S3.** Functional connectivity change during extinction learning correlates with neural signals during memory recall test, for the network component identified with HC vs. PTSD. **A.** The functional connectivity change positively correlates with vmPFC activation during memory recall within the HC group. **B.** The connectivity change positively correlates with functional connectivity during memory recall within the HC group.


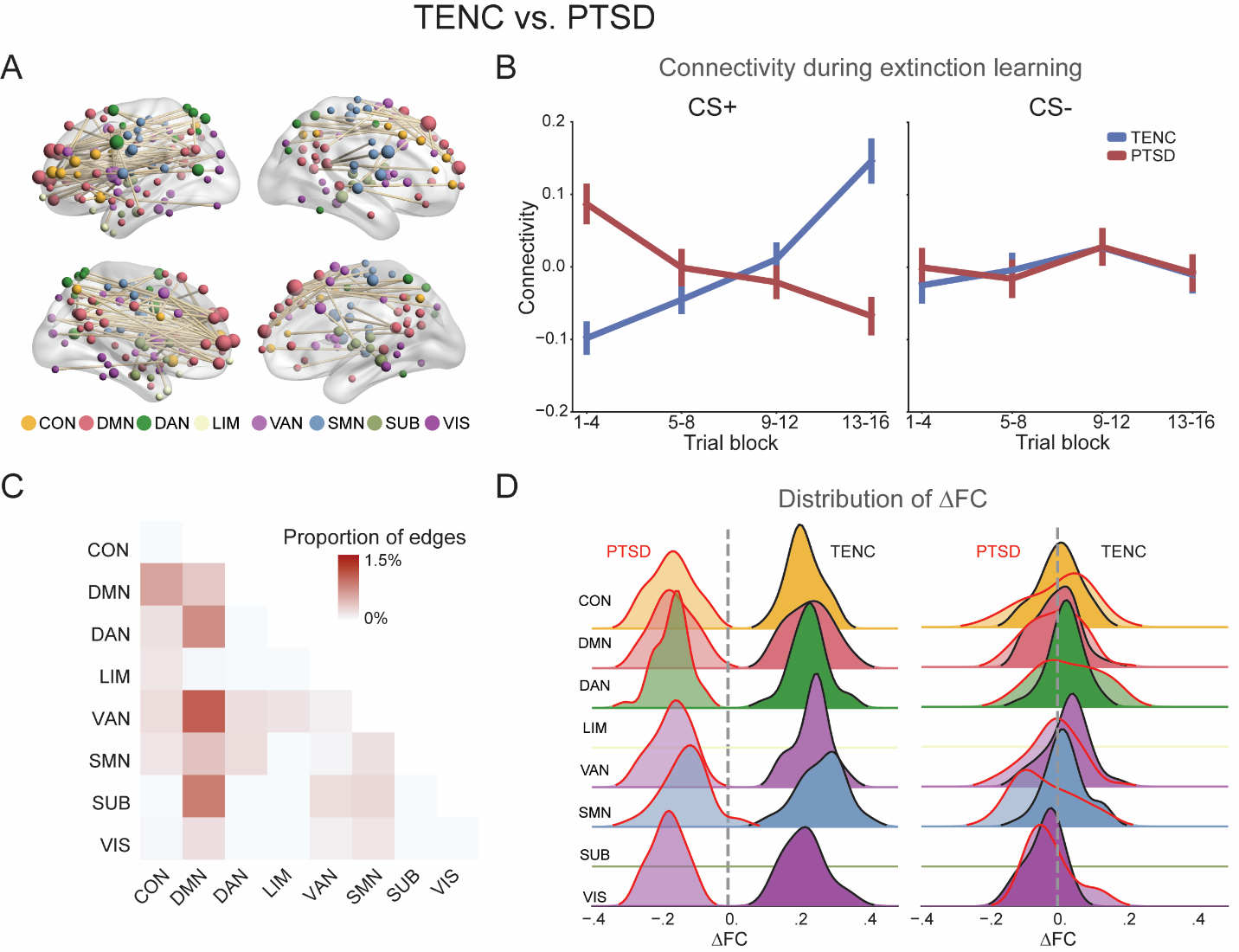


**Supplementary Figure S4.** Abnormal dynamic functional connectivity in the PTSD group during extinction learning when compared with TENC. **A.** The network component that showed significant difference between TENC and PTSD groups. **B.** Mean functional connectivity of the identified network component during extinction learning, separately for CS+ processing or CS- processing. **C.** The proportion of significant edges within or between the 8 subnetworks. A darkly shaded cell indicates that the connections of that network pair (indexed from x- and y-axis) were extensively impaired. **D.** Distribution of mean connectivity change (ΔFC, late minus early extinction learning) with each of the 8 subnetworks during CS+ processing or CS- processing. TENC: trauma-exposed non-PTSD controls.


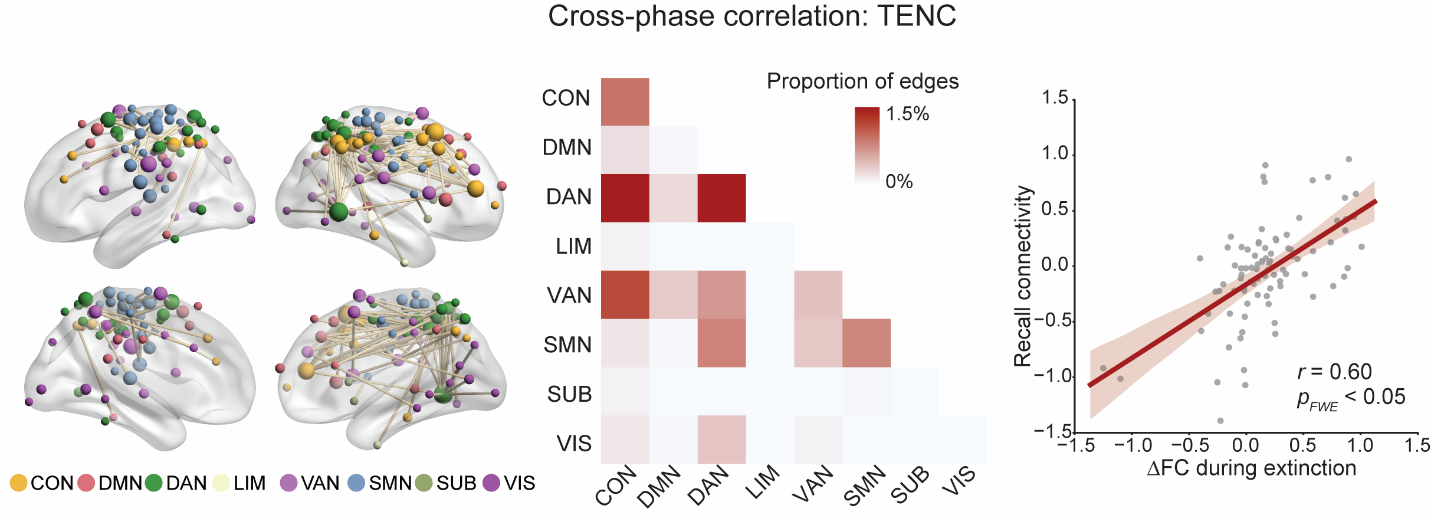


**Supplementary Figure S5.** Functional connectivity change during extinction learning positively correlates with functional connectivity during memory recall test within the TENC group. The network component was identified with TENC vs. PTSD.


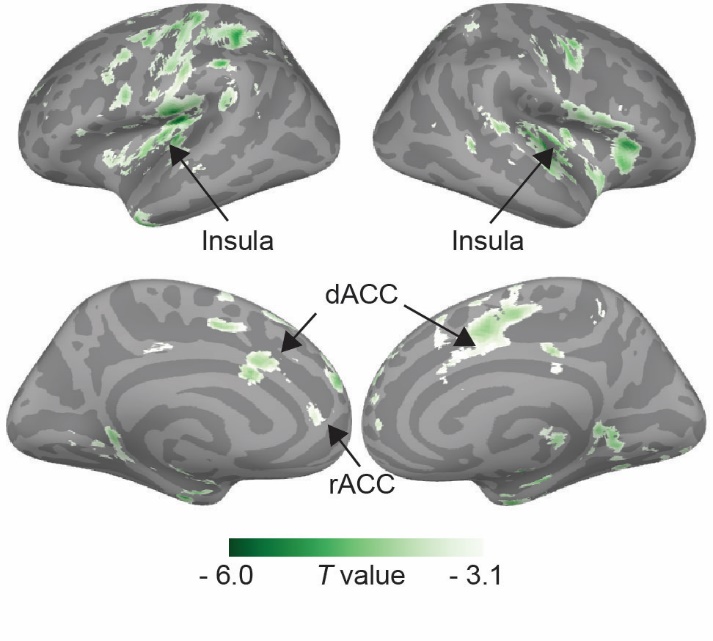


**Supplementary Figure S6.** Brain regions showed significant negative correlation between connectivity change and activation change during extinction learning in the AX group.


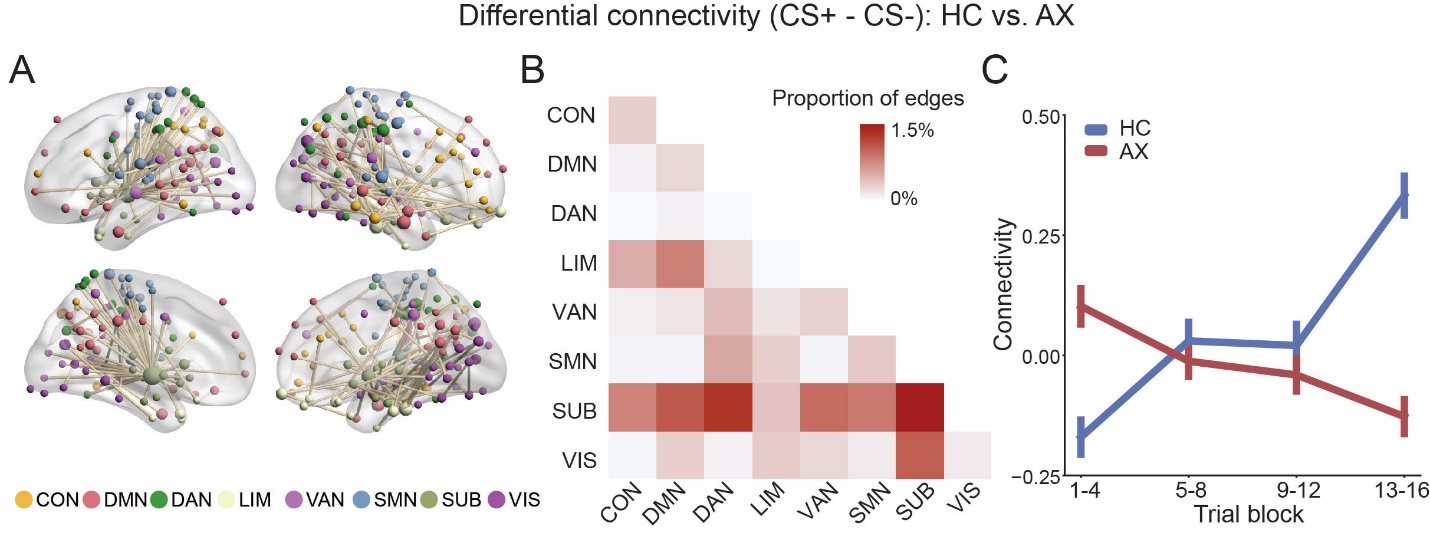


**Supplementary Figure S7.** Abnormal change of differential connectivity in the AX group during extinction learning when compared with HC. **A.** The network component that showed significant difference between HC and AX groups. **B.** The proportion of significant edges within or between the 8 subnetworks. A darkly shaded cell indicates that the connections of that network pair (indexed from x- and y-axis) were extensively impaired. **C.** Mean differential connectivity (CS+ - CS-) of the identified network component during extinction learning, separately for HC and AX.
